# A novel role of IGFBP5 in the migration, invasion and spheroids formation induced by IGF-I and insulin in MCF-7 breast cancer cells

**DOI:** 10.1007/s10549-024-07397-5

**Published:** 2024-06-19

**Authors:** Karem Rodríguez-Rojas, Pedro Cortes-Reynosa, Pablo Torres-Alamilla, Nínive Rodríguez-Ochoa, Eduardo Perez Salazar

**Affiliations:** Departamento de Biologia Celular. Centro de Investigacion y de Estudios Avanzados del Instituto Politecnico Nacional, Av. IPN # 2508, 07360 Mexico City, Mexico

**Keywords:** Breast cancer, IGFBP5, IGF-I, Insulin, MCF-7, Spheroids

## Abstract

**Purpose:**

The insulin-like growth factor (IGF) system includes IGF-I, IGF-II insulin and their membrane receptors. IGF system also includes a family of proteins namely insulin-like growth factor-binding proteins (IGFBPs) composed for six major members (IGFBP-1 to IGFBP6), which capture, transport and prolonging half-life of IGFs. However, it has been described that IGFBPs can also have other functions.

**Methods:**

IGFBP5 expression was inhibited by shRNAs, migration was analyzed by scratch-wound assays, invasion assays were performed by the Boyden chamber method, spheroids formation assays were performed on ultra-low attachment surfaces, expression and phosphorylation of proteins were analyzed by Western blot.

**Results:**

IGFBP5 is a repressor of IGF-IR expression, but it is not a repressor of IR in MCF-7 breast cancer cells. In addition, IGFBP5 is a suppressor of migration and MMP-9 secretion induced by IGF-I and insulin, but it does not regulate invasion in MCF-7 cells. IGFBP5 also is a repressor of MCF-7 spheroids formation. However treatment with 340 nM rescues the inhibitory effect of IGFBP in the MCF-7 spheroids formation.

**Conclusion:**

IGFBP5 regulates IGF-IR expression, migration and MMP-9 secretion induced by IGF-I and/or insulin, and the spheroids formation in MCF-7 breast cancer cells.

**Supplementary Information:**

The online version contains supplementary material available at 10.1007/s10549-024-07397-5.

## Introduction

Data from Globocan show that breast cancer is the most prevalent neoplasia and the top cause of deaths associated with cancer in women worldwide [1]. The highest incidence for breast cancer occurs in high-income countries, however breast cancer incidence has been growing in the last years in low- to middle-income countries, and they have the highest mortality [2, 3]. Molecular subtypes for breast cancer consider the expression of Ki-67, estrogen receptor (ER), progesterone receptor (PR) and the human epidermal growth factor receptor 2 (HER2) [4, 5]. Luminal A subtype expresses ER and PR; luminal B subtype is ER+ , PR ± with a high expression of Ki67; Her2 subtype is Her2 + , and a high expression of Ki67; and triple negative breast cancer (TNBC) subtype is ER−, PR−, Her2− and a high expression of Ki67 [6].

The insulin-like growth factor (IGF) system comprises IGF-I, IGF-II, insulin, and their membrane receptors, which include IGF-I receptor (IGF-IR), IGF-II receptor (IGF-IIR), insulin receptor (IR) A, and IR B [7]. The IGF system plays an important role in lipid and glucose metabolism, proliferation, invasion and differentiation for a variety of tissues [7]. Moreover, IGF/IGFR system includes a highly conserved family of proteins named insulin-like growth factor-binding proteins (IGFBPs). IGFBPs are a familly of proteins constituted of six major members named IGFBP1 to IGFBP6, which have a high affinity for IGF-I and IGF-II, but they do not present affinity for insulin. IGFBPs are secreted by liver and are responsible for capture, transport and prolonging the half-life of IGFs in bloodstream. In the target tissues, the IGFBPs are degradated by specific proteases and then IGFs are released and mediate a variety of signals via activation of IGF-IR and/or IRs [8].

IGFBP5 is a 31–32 kDa glycosylated protein constituted of three domains: an N-terminal domain (1–80 amino acids), a linker domain (81–170) and a C-terminal domain (171–252 amino acids) [9, 10]. The linker domain is the substrate for a variety of proteases including ADAM9, MMP-2, PSA, thrombin and PAPP-A, whereas the 40–92 amino acids in the N-terminal domain can be phosphorylated and mediate the IGF-I binding site, however the maximum inhibitory activity requires the C-terminal domain [11, 12]. IGFBP5 is expressed in a variety of tissues including bone, muscle, testis, ovary, kidney and breast. Breast cancer cells release IGFBP5, IGF-I and insulin, and they regulate tumor progression in a paracrine and endocrine manner [13].

Focal adhesion kinase (FAK) is a nonreceptor protein tyrosine kinase ubiquitously expressed and activated by a variety of receptors including integrins, G-protein-coupled receptors and cytokine and growth factors receptors. It regulates a variety of cellular processes including proliferation, migration, adhesion and survival [14, 15]. The structure of FAK is composed of three domains: an N-terminal FERM domain, a central kinase domain and the C-terminal focal adhesion targeting (FAT) domain. FAK is activated by autophosphorylation at tyrosine (Tyr)-397, which creates the formation of a high affinity binding site for Src SH2 domain, and the formation of FAK-Src complex throught binding of Src at Try-397 of FAK. The FAK-Src complex promotes that Src phosphorylates FAK at Tyr-576 and Tyr-577 in the activation loop and it promotes the maximal catalityc activity of FAK [14, 16]. FAK is also phosphorylated at multiple serine (Ser) residues within its C-terminal domain, such as Ser-722, Ser-843, Ser-846 and Ser-910, which are in close proximity to the domains that mediate the protein–protein interactions for FAK [17, 18]. It has been suggested that phosphorylation of these Ser residues regulates the assembly of FAK signaling complexes, such as the FAK-p130^Cas^ complex [16, 19].

We demonstrate that IGFBP5 is a repressor of IGF-IR expression, but is not a repressor of IR expression in MCF-7 breast cancer cells. In addition, IGFBP5 is a suppressor of migration and MMP-9 secretion induced by IGF-I and insulin. However IGFBP5 does not regulate invasion in MCF-7 cells. IGFBP5 is also a repressor of MCF-7 cells spheroids formation on ultra-low attachment surfaces, but treatment with 340 nM insulin rescues the inhibitory effect of IGFBP5.

## Materials and methods

### Materials

IGFBP5 human recombinant protein, IGF-I and insulin were from Sigma-Aldrich Chemicals Co (St. Louis, MO). IGFBP5 antibody (Ab), insulin receptor β Ab, FAK Ab and lentiviral shRNA vectors against IGFBP5 were from Santa Cruz Biotechnology (St. Cruz, CA). IGF-I receptor β Ab and phosphospecific Ab to Tyr-397 of FAK (anti-p-FAK) were from Cell Signaling Technology (Beverly, MA). Phosphospecific Ab to Ser-910 of FAK (anti-p-FAK Ser-910) was from ThermoFisher Scientific, Invitrogen (Waltham, MA). β-actin Ab was from R&D systems (Minneapolis, MN).

### Cell culture

MCF-7 and MDA-MB-231 breast cancer cells were obtained from American Type Culture Collection and they were authenticated via short tandem repeat DNA profiling. MCF-7 and MDA-MB-231 cells were cultured in Dulbecco’s Modified Eagle’s Minimum (DMEM) containing 5% fetal bovine serum (FBS) and antibiotics in a humidified atmosphere containing 5% CO_2_ and 95% air at 37 °C. For experimental procedures, MCF-7 and MDA-MB-231 cells were FBS starved for 18 h in DMEM before treatment.

### Silencing of IGFBP5 expression with shRNAs

Lentiviral shRNA vectors targeting human IGFBP5 were used for generation of stable knockdown of IGFBP5 in MCF-7 and MDA-MB-231 cells, according the manufacturer´s guidelines. Transfected MCF-7 and MDA-MB-231 cells were selected by their resistance to puromycin (5 µg/ml).

### Western blotting (WB)

WB was performed as described previously using 30 µg of total protein [20]. Autoradiograms were scanned and bands were quantified using the ImageJ software v1.52u (NIH, USA).

### Scratch-wound assay

Cultures of MCF-7 and MDA-MB-231 cells were treated for 2 h with 12 µM mitomycin C to inhibit cell proliferation. Cultures were scratch-wounded, washed twice with PBS and re-fed with DMEM without or with 50 ng/ml IGF-I, 100 and 340 nM insulin for 48 h at 37 °C. At the end of incubation, cells were fixed with 4% paraformaldehyde for 15 min and stained with 0.5% crystal violet for 10 min. Images from ≥ 3 fields per experimental condition were acquired using an inverted microscope coupled to a camera and analyzed using the ImageJ software v1.52u (NIH, USA).

### Invasion assay

Invasion assays were performed by the modified Boyden chamber method using 24-well plates with 12 cell culture inserts with 8 µm pore size as described previously [21].

### Spheroid formation assay on ultra-low attachment surface

Confluent cultures of MCF-7 cells (80%) were harvested with trypsin and pipetted to form a single cell suspension. Trypsin was inactivated by addition of DMEM with 5% FBS and cells were obtained by centrifugation. Cells were quantified and seeded at 5000 cells per well in six-well ultra-low attachment cluster dishes (Costar, Inc). Spheroids were cultured in DMEM with 1% FBS in the absence or presence of 50 ng/ml IGF-I, 340 nM insulin and 10% FBS for 9 days, which were refreshed every 3 days. Spheroids number and area were quantified using an inverted microscope coupled to a camera and the ImageJ software v1.52 (NIH, USA).

### Statistical analysis

**Fig. 1:**
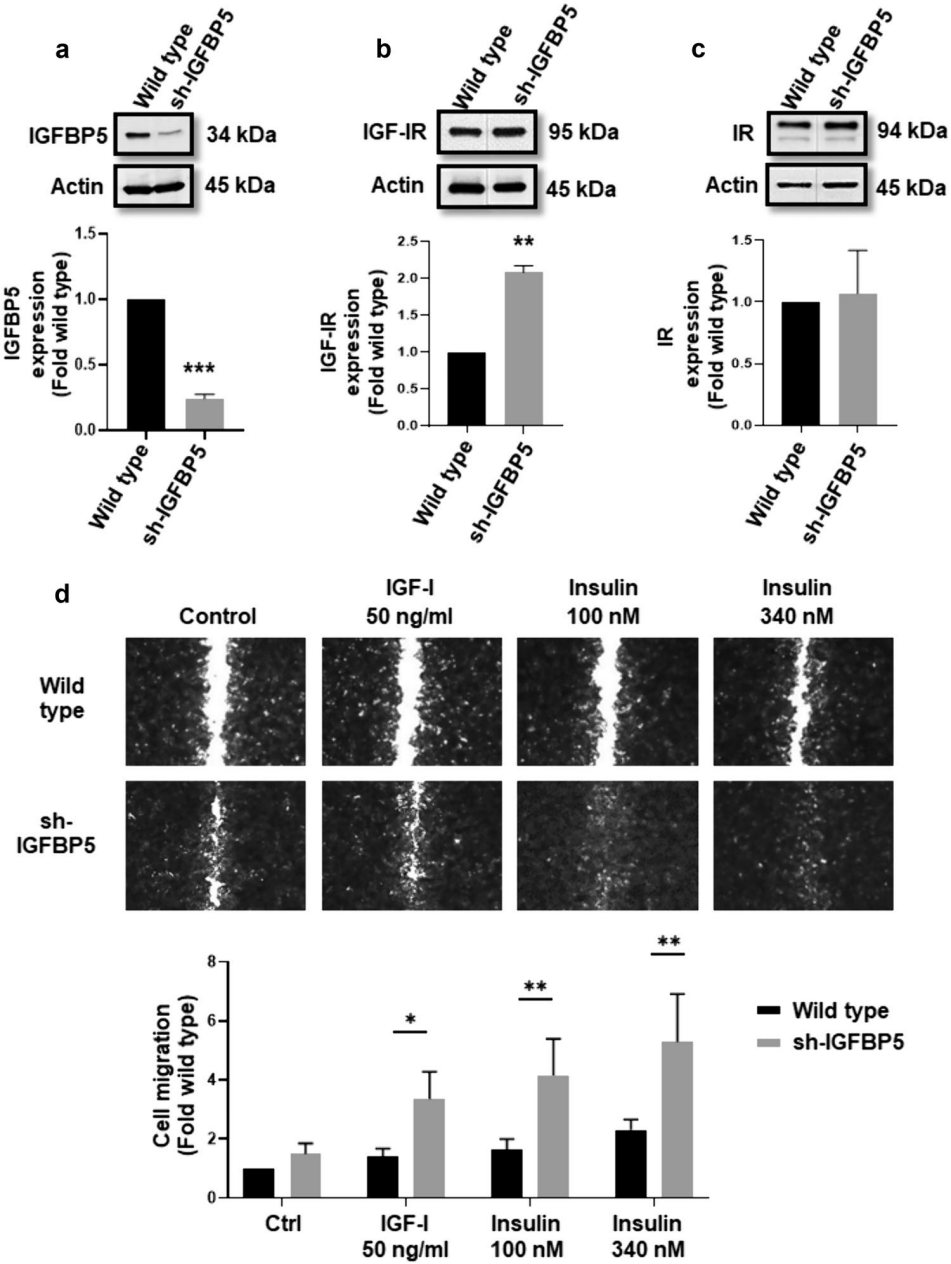
IGFBP5 regulates IGF-IR expression and migration in MCF-7 breast cancer cells. **a–c** Lysates from wild type MCF-7 cells and sh-IGFBP5 MCF-7 cells were analyzed by WB with anti-IGFBP5 Ab, anti-IGF-IR Ab, anti-IR Ab and anti-actin Ab. **d** Confluent cultures of wild type MCF-7 cells and sh-IGFBP5 MCF-7 cells were scratched and untreated and treated with 50 ng/ml IGF-I, 100 and 340 nM insulin. Graphs are the mean ± S.D. and indicate the fold of protein expression (IGFBP5, IGF-IR, IR) or migration above control value. *P < 0.05, **P < 0.01, ***P < *0.001*

Results are expressed as mean ± S.D. of at least three independent experiments. Data corresponding to experiments of Figs. 1a, b, c and 5c were analyzed by Student’s t-test. The other data were analyzed by two-way ANOVA statistical test and Dunnett’s post hoc test. A statistical probability of *P* ≤ *0.05* was considered significant. Asterisks denote comparisons made to the control.

## Results

### IGFBP5 inhibits migration induced by IGF-I and insulin in MCF-7 breast cancer cells

**Fig. 2:**
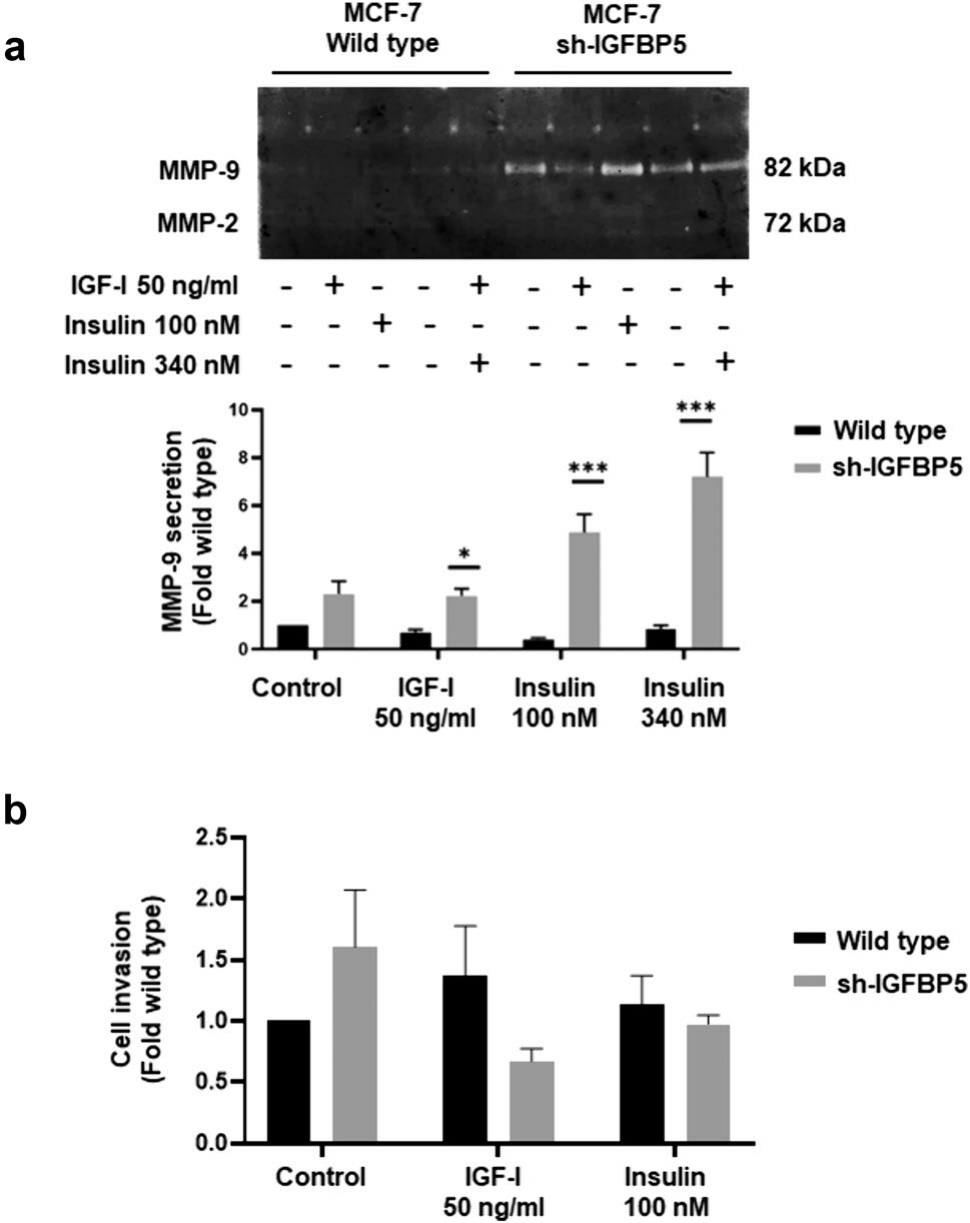
IGFBP5 inhibits MMP-9 secretion induced by IGF-I and insulin. **a** Wild type MCF-7 cells and sh-IGFBP5 MCF-7 cells were treated with 50 ng/ml IGF-I, 100 and 340 nM insulin for 48 h and then conditioned media were obtained. MMP-2 and MMP-9 secretion was analyzed by gelatin-substrate gels. **b** Invasion assays were performed using the Boyden chamber method with wild type MCF-7 cells and sh-IGFBP5 MCF-7 cells treated with 50 ng/ml IGF-I, 100 nM insulin for 48 h. Graphs are the mean ± S.D. and indicate the fold of MMP-9 secretion or invasion above control value. *P < 0.05, ***P < *0.001*

We determined whether IGFBP5 induced migration in MCF-7 cells. Migration assays were performed with MCF-7 cells treated with 50–200 ng/ml IGFBP5. Findings showed that treatment with 50 and 100 ng/ml IGFBP5 did not induce migration, however treatment with 200 ng/ml IGFBP5 inhibited basal migration (35%) in MCF-7 cells (Fig. 1Sa). Interestingly, IGFBP5 induced migration at 100 ng/ml (45%) in TNBC MDA-MB-231 cells (Fig. 2Sa).

Since, IGFBP5 did not induce migration, we studied whether IGFBP5 inhibited migration induced by IGF-I and insulin. IGFBP5 expression was inhibited in MCF-7 cells by using IGFBP5 shRNA lentiviral particles. Cells were lysed and analyzed by WB with anti-IGFBP5 Ab, anti-IGF-IR Ab, anti-IR Ab and anti-actin Ab as loading control. As illustrated in Fig. 1a, MCF-7 cells transfected with IGFBP5 shRNA lentiviral particles (sh-IGFBP5 MCF-7) showed a significant inhibition of IGFBP5 expression (76%). However, inhibition of IGFBP5 expression induced an increase of IGF-IR expression (207%), but it did not modify IR expression in MCF-7 cells (Fig. 1b and c).

Next, migration assays were performed with wild type MCF-7 cells and sh-IGFBP5 MCF-7 cells treated with 50 ng/ml IGF-I, 100 and 340 nM insulin. Our results showed that treatment with 50 nM IGF-I, 100 and 340 nM insulin induced higher migration in sh-IGFBP5 MCF-7 cells (193%, 250% and 296% respectively) than in wild type MCF-7 cells (Fig. 1d). Interestingly, treatment with 50 nM IGF-I did not induce migration in wild type MDA-MB-231 cells and MDA-MB-231 cells transfected with IGFBP5 shRNA lentiviral particles (sh-IGFBP5 MDA-MB-231). However, treatment with 100 nM insulin induced inhibition of migration in sh-IGFBP5 MDA-MB-231 cells (32%) compared with wild type MDA-MB-231 cells, whereas 340 nM insulin induced higher migration in sh-IGFBP5 MDA-MB-231 cells (108%) than in wild type MDA-MB-231 cells (Fig. 2Sb and c).

### IGFBP5 inhibits MMP-9 secretion induced by IGF-I

We determined whether IGFBP5 inhibited MMP-2 and MMP-9 secretion. Conditioned media from wild type MCF-7 cells and sh-IGFBP5 MCF-7 cells treated with 50 ng/ml IGF-I, 100 and 340 nM insulin were analyzed by gelatin zymography. As illustrated in Fig. 2a, treatment with 50 ng/ml IGF-I, 100 and 340 nM insulin induced a higher secretion of MMP-9 in sh-IGFBP5 MCF-7 cells (150%, 444% and 636% respectively) than in wild type MCF-7 cells. In contrast, treatment with IGF-I and insulin did not induce an increase of MMP-2 secretion.

Invasion assays were performed with wild type MCF-7 cells and sh-IGFBP5 MCF-7 cells treated with 50 ng/ml IGF-I and 100 nM insulin. Our findings showed that treatment with 50 ng/ml IGF-I and 100 nM insulin did not induce invasion in wild type MCF-7 cells and sh-IGFBP5 MCF-7 cells (Fig. 2b).

### IGFBP5 inhibits FAK phosphorylation at Ser-910 induced by insulin

**Fig. 3:**
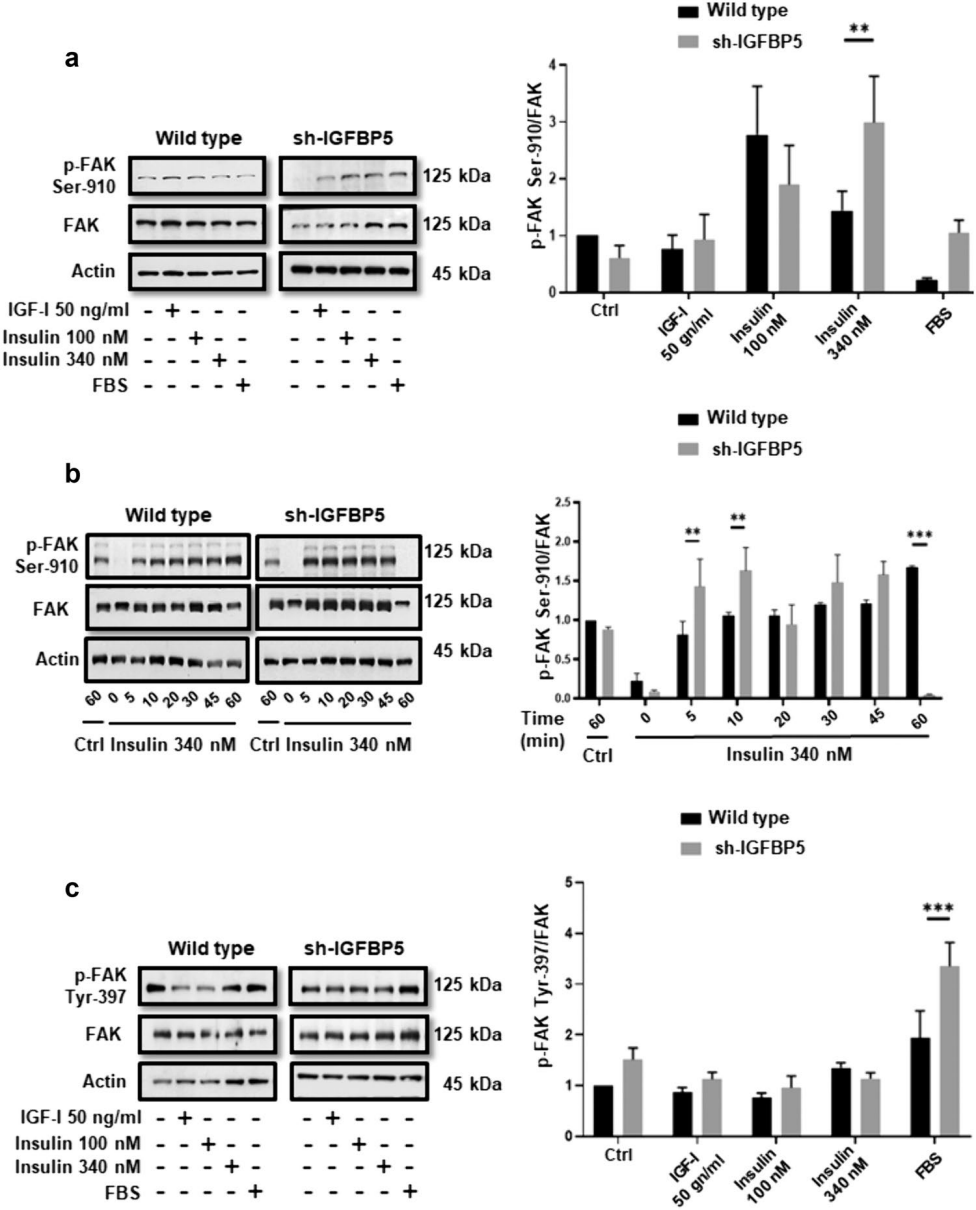
IGFBP5 inhibits FAK phosphorylation at Ser-910 induced by insulin. **a** Wild type MCF-7 cells and sh-IGFBP5 MCF-7 cells were treated with 50 ng/ml IGF-I, 100 and 340 nM insulin for 10 min and lysed. **b** Wild type MCF-7 cells and sh-IGFBP5 MCF-7 cells were untreated and treated with 340 nM insulin for 5, 10, 20, 30, 45 and 60 min and lysed. Cell lysates were analyzed by WB with anti-p-FAK Ser 910 Ab, anti-FAK Ab and anti-actin Ab. One control of untreated cells and incubated for 60 min was included. **c** Wild type MCF-7 cells and sh-IGFBP5 MCF-7 cells were treated with 50 ng/ml IGF-I, 100 and 340 nM insulin for 20 min and lysed. Cell lysates were analyzed by WB with anti-p-FAK Ab. anti-FAK Ab and anti-actin Ab. One control of FBS was included. Graphs are the mean ± S.D. and indicate the fold of FAK phosphorylation at Ser 910 and Tyr 397 above control value. **P < 0.01, ***P < *0.001*

We determined whether IGFBP5 inhibited FAK phosphorylation at Ser-910. Cell lysates from wild type MCF-7 cells and sh-IGFBP5 MCF-7 cells treated with 50 ng/ml IGF-I, 100 and 340 nM insulin for 10 min were analyzed by WB with anti-p-FAK Ser-910 Ab. As illustrated in Fig. 3a, treatment with 340 nM insulin induced a higher FAK phosphorylation at Ser-910 in sh-IGFBP5 MCF-7 cells (140%) than in wild type MCF-7 cells (Fig. 3a).

Next, we determined whether treatment with 340 nM insulin induced FAK phosphorylation at Ser-910 in a time-dependent manner. Cell lysates from wild type MCF-7 cells and sh-IGFBP5 MCF-7 cells treated with 340 nM insulin for various times were analyzed by WB with anti-p-FAK Ser-910 Ab. As illustrated in Fig. 3b, treatment with 340 nM insulin for 5 and 10 min induced a higher FAK phosphorylation at Ser-910 in sh-IGFBP5 MCF-7 cells (61% and 57% respectively) than in wild type MCF-7 cells. However, treatment with 340 nM insulin for 60 min induced a higher FAK phosphorylation at Ser-910 in wild type MCF-7 cells than in sh-IGFBP5 MCF-7 cells.

We also analyzed whether IGF-I and insulin induced an increase of FAK phosphorylation at Tyr-397. Cell lysates from wild type MCF-7 cells and sh-IGFBP5 MCF-7 cells treated with 50 ng/ml IGF-I, 100 and 340 nM insulin for 20 min were analyzed by WB with anti-p-FAK Ab. Our findings showed that treatment with 50 ng/ml IGF-I, 100 and 340 nM insulin did not induce an increase of FAK phosphorylation at Tyr-397 in wild type MCF-7 cells and sh-IGFBP5 MCF-7 cells (Fig. 3c).

### IGFBP5 inhibits IGF-IR expression in MCF-7 cells

**Fig. 4:**
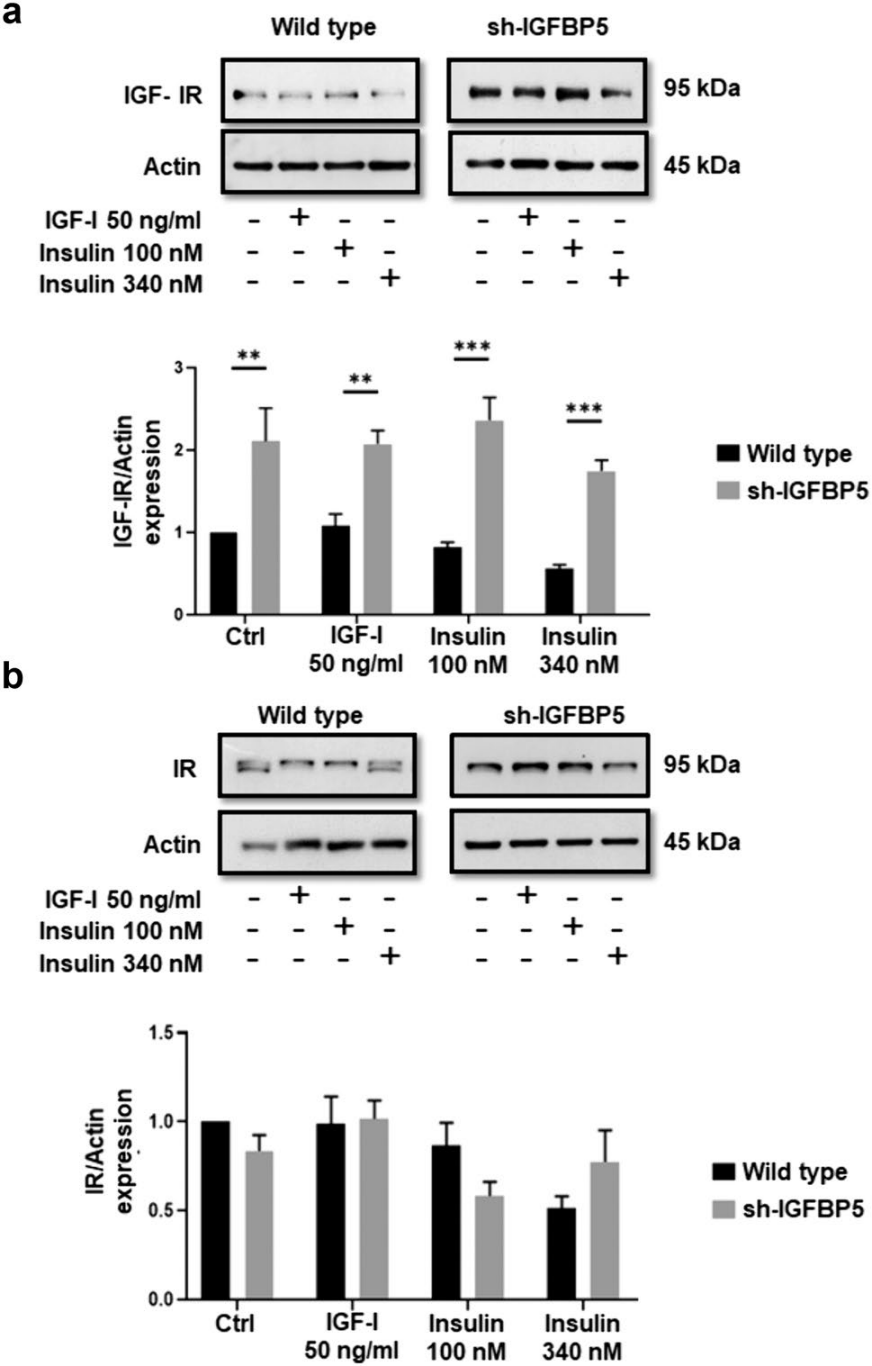
IGFBP5 inhibits IGF-IR expression. **a** and **b** Wild type MCF-7 cells and sh-IGFBP5 MCF-7 cells were treated with 50 ng/ml IGF-I, 100 and 340 nM insulin for 2 h and lysed. Cell lysates were analyzed by WB with anti-IGF-IR Ab, anti-IR Ab and anti-actin Ab. Graphs are the mean ± S.D. and indicate the fold of IGF-IR or IR above control value.**P < 0.01, ***P < *0.001*

We determined whether IGFBP5 inhibited IGF-IR and IR expression in response to IGF-I and insulin. Cell lysates from wild type MCF-7 cells and sh-IGFBP5 MCF-7 cells treated with 50 ng/ml IGF-I, 100 and 340 nM insulin were analyzed by WB with anti-IGF-IR Ab and anti-IR Ab. Findings showed that inhibition of IGFBP5 expression induced a higher expression of IGF-IR in untreated sh-IGFBP5 MCF-7 cells (110%) than in untreated wild type MCF-7 cells. Interestingly, treatment with 50 ng/ml IGF-I, 100 and 340 nM insulin induced an increase of IGF-IR expression in sh-IGFBP5 MCF-7 cells (99%, 154% and 119% respectively) compared with wild type MCF-7 cells (Fig. 4a).

Our findings also demonstrated that inhibition of IGFBP5 expression did not modify IR expression in wild type MCF-7 cells and sh-IGFBP5 MCF-7 cells untreated and treated with 50 ng/ml IGF-I, 100 and 340 nM insulin (Fig. 4b).

### IGFBP5 mediates the formation of spheroids on ultra-low attachment surface

**Fig. 5:**
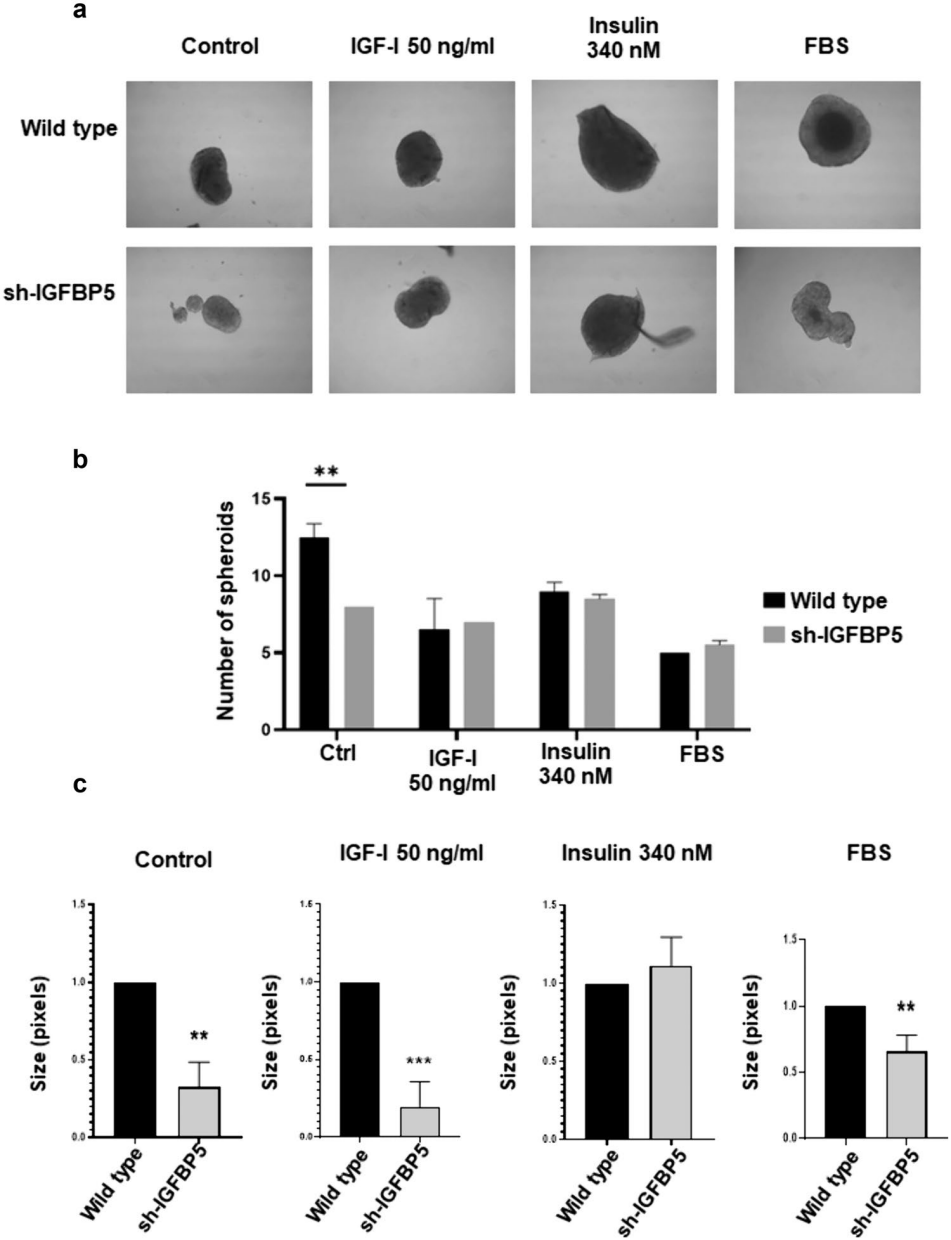
Role of IGFBP5 in the spheroids formation induced by IGF-I and insulin. **a** Representative images of spheroids using wild type MCF-7 cells and sh-IGFBP5 MCF-7 cells untreated and treated with 50 ng/ml IGF-I, 340 nM insulin and 10% FBS. **b** Number of spheroids using wild type MCF-7 cells and sh-IGFBP5 MCF-7 cells untreated and treated with 50 ng/ml IGF-I, 340 nM insulin and 10% FBS. **c** Size of spheroids using wild type MCF-7 cells and sh-IGFBP5 MCF-7 cells untreated and treated with 50 ng/ml IGF-I, 340 nM insulin and 10% FBS. Graphs are the mean ± S.D. and indicate the spheroids number or size of spheroids above wild type value. **P < *0.01*

We studied the role of IGFBP5 in the formation of spheroids. Spheroid formation assays were performed on ultra-low attachment surfaces using wild type MCF-7 cells and sh-IGFBP5 MCF-7 cells untreated and treated with 50 ng/ml IGF-I and 340 nM insulin. Findings showed that total number of spheroids is higher in untreated wild type MCF-7 cells (100%) than in untreated sh-IGFBP5 MCF-7 cells (64%). However, treatment with 50 ng/ml IGF-I and 340 nM insulin induced the formation of a similar number of spheroids in wild type MCF-7 cells and sh-IGFBP5 MCF-7 cells (Fig. 5a and b).

We also analyzed the size of spheroids. Findings showed that inhibition of IGFBP5 expression induced the formation of smaller spheroids in untreated and treated with 50 ng/ml IGF-I sh-IGFBP5 MCF-7 cells (65% and 83% respectively) compared with wild type MCF-7 cells (100%). However, treatment with 340 nM insulin induced the formation of spheroids with similar size in wild type MCF-7 cells and sh-IGFBP5 MCF-7 cells (Fig. 5c).

## Discussion

IGFBP5 has a variety of functions including proliferation during breast maturation. In addition, IGFBP5 may be a promoter or suppressor of tumor growth in a variety of cancers, including breast cancer [22–24]. However, the role of IGFBP5 in cellular processes that mediate tumor growth and metastasis through the IGF/IGFR system in breast cancer has not been studied in detail.

IGFBP5 enhances migration and proliferation of mesenchymal stem cells in the presence of TNFα, and induces migration in primary human lung fibroblasts and peripheral blood mononuclear cells [25, 26]. Findings demonstrate that IGFBP5 does not induce migration in MCF-7 breast cancer cells, but it induces migration in TNBC MDA-MB-231 cells. Since, MCF-7 cells express ER and PR, whereas MDA-MB-231 cells do not express ER, PR and Her2. We propose that IGFBP5 mediates different biological process depending on the expression of PR, ER and Her2 receptors in breast cancer.

The IGF system is composed of IGF-I, IGF-II, insulin and their receptors (IGF-IR, IGF-IIR and IR) [7, 27]. Interestingly, our findings show that inhibition of IGFBP5 expression induces an increase of IGF-IR expression in MCF-7 cells. In agreement with our findings, inhibition of IGFBP3 expression induces an increase of IGF-IR expression in human telomerized corneal epithelial cells [28]. We propose that IGFBP5 induces the activation of specific receptors that inhibit IGF-IR expression in MCF-7 cells. Supporting our proposal, IGFBP5 has specific tyrosine kinase receptors in a variety of cells [29].

Metastasis is a complex process that involves the formation of distal secondary tumors, and is mediated by release of cells from primay tumor, migration, invasion, intravasation, extravasion, evasion of immune surveillance and regulation of tissue microenvironment [30, 31]. Particularly, MMP-2 and MMP-9 degrade type IV collagen, which is the main component of basement membranes, such as basement membranes of epithelial cells in the lobules and ducts of mammary glands [32]. We study the role of IGFBP5 in some cell biological processes that mediate the tumor growth and metastasis process. In this study MCF-7 cells are treated with 50 ng/ml IGF-I, because it induces the maximum of migration in MCF-7 cells (Fig. 1Sb). Moreover, MCF-7 cells are treated with 100 and 340 nM insulin, because 100 nM insulin induces proliferation in MCF-7 cells, whereas 340 nM insulin induces migration in MCF-7 cells and an increase of IR expression in MCF-7 cells pretreated with linoleic acid [33, 34]. Our findings show that IGF-I and insulin induce a higher migration and MMP-9 secretion in sh-IGFBP5 MCF-7 cells than wild type MCF-7 cells. Therefore, we demonstrate that IGFBP5 is a repressor of migration and MMP-9 secretion induced by IGF-I and insulin in MCF-7 cells. Interestingly, IGFBP5 is a repressor of migration in MDA-MB-231 cells treated with 340 nM insulin, but it is not a repressor when the cells are treated with 100 nM insulin and 50 mg/ml IGF-I. We propose that IGFBP5 regulates negatively the expression and/or activation of IGF-IR, IR and then the signal transduction pathways that mediate migration and MMP-9 secretion induced by IGF-I and insulin in MCF-7 cells. Supporting our proposal, we demonstrate here that inhibition of IGFBP5 expression induces and increase of IGF-IR expression in MCF-7 cells. In addition, IGFBP5 increases adhesion to vitronectin through the interaction between IGFBP5 and α2β1, and it inhibits migration via Cdc42 and the activation of integrin-linked kinase and Akt in MCF-7 cells [24]. Moreover, overexpression of IGFBP5 in MDA-MB-435 breast cancer cells promotes a lower cell growth rate and motility than wild type MDA-MB-435 cells and the localization of IGFBP5 in the nucleus [35]. Our findings also support the proposal that IGFBP5 regulates specific biological processes, such as migration, depending on the expression of PR, ER and Her2 receptors.

FAK is overexpressed in a variety of human cancers, including breast cancer [36]. Moreover, FAK is localized in focal adhesions and plays an important role in migration and invasion [14, 16]. Our findings demonstrate that treatment with 340 nM insulin induces a higher FAK phosphorylation at Ser-910 in sh-IGFBP5 MCF-7 cells than in wild type MCF-7 cells. We propose that IGFBP5 is an inhibitor of migration induced by 340 nM insulin through FAK phosphorylation at Ser-910 in MCF-7 cells. Supporting our proposal, FAK phosphorylation at Ser-910 is essential for migration of B16F10 murine melanoma cells and for lung metastasis in a murine model of metastasis using B16F10A melanoma cells and C57BL/6J mice [37].

FAK activation is mediated by its autophosphorylation at Tyr-397 [16]. Our findings demonstrate that wild-type MCF-7 cells and sh-IGFBP5 MCF-7 cells express basal levels of FAK phosphorylation at Tyr-397, and treatment for 20 min with 50 ng/ml IGF-I, 100 and 340 nM insulin do not induce an increase of FAK phosphorylation at Tyr-397. In agreement with our findings, angiotensin II, lysophosphatidic acid, phorbol-12,13-dibutyrate and epidermal growth factor induce FAK phosphorylation at Ser-910 through an ERK-dependent pathway, but they do not induce an increase of FAK phosphorylation at Tyr-397 in IEC-18 intestinal epithelial cells [38]. We propose that IGFBP5 inhibits FAK phosphorylation at Ser-910 induced by 340 nM insulin, and that FAK phosphorylation at Ser-910 represses FAK phosphorylation at Tyr-397 and then cell spreading and migration in MCF-7 cells. Supporting our proposal, FAK phosphorylation at Ser-843 inhibits Tyr-397 phosphorylation, cell spreading and migration [39].

In order to substantiate our findings, we performed spheroids formation assays on ultra-low attachment surface using wild type MCF-7 cells and sh-IGFBP5 MCF-7 cells stimulated with 50 ng/ml IGF-I and 340 nM insulin. Our findings show that number of spheroids using untreated sh-IGFBP5 MCF-7 cells is lower than number of spheroids using untreated wild type MCF-7 cells. Moreover, treatment with IGF-I and insulin promote the formation of a similar number of spheroids using wild type MCF-7 cells and sh-IGFBP5 MCF-7 cells.

Interestingly, untreated wild type MCF-7 cells are able to form larger spheroids than untreated sh-IGFBP5 MCF-7 cells, and it is not affected by stimulation with IGF-I. However, treatment with 340 insulin promotes formation of spheroids with similar size using wild type MCF-7 cells and sh-IGFBP5 MCF-7 cells. We propose that IGFBP5 plays a role in the proliferation and then in the growth of MCF-7 cells spheroids. However, insulin is able to replace the role of IGFBP5 in the growth of MCF-7 spheroids. Supporting our proposal, proteome analysis of canine mammary tumors demonstrate that IGFBP5 is highly expressed in the different stage of mammary tumors [40].

## Conclusion

We demonstrate a novel function of IGFBP5 in the regulation of IGF-IR expression, migration and MMP-9 secretion induced by IGF-I and/or insulin, and in the spheroids formation in MCF-7 breast cancer cells.

## Supplementary Information

Below is the link to the electronic supplementary material.

Supplementary file1 (TIF 151 KB) Figure 1S Role of IGFBP5 and IGF-I in the migration of MCF-7 and MDA-MB-231 cells. a Confluent cultures of MCF-7 cells were scratched and treated with 50, 100 and 200 ng/ml IGFBP5 for 48 h. b Confluent cultures of MCF-7 cells were scratched and treated with 10, 20, 50 and 100 ng/ml IGF-I for 48 h. Graph is the mean ± S.D. and indicates the fold of migration above control value. **P < 0.01, ***P < 0.001

Supplementary file2 (TIF 212 KB) Figure 2S IGFBP5 induces migration in MDA-MB-231 breast cancer cells. a Confluent cultures of MDA-MB-231 cells were scratched and treated with 50, 100, 200 and 500 ng/ml IGFBP5 for 48 h. b Lysates from wild type MDA-MB-231 cells and sh-IGFBP5 MDA-MB-231 cells were analyzed by WB with anti-IGFBP5 Ab and anti-actin Ab. c Confluent cultures of wild type MDA-MB-231 cells and sh-IGFBP5 MDA-MB-231 cells were scratched and untreated and treated with 50 ng/ml IGF-I, 100 and 340 nM insulin. Graphs are the mean ± S.D. and indicate the fold of migration above control value. *P<0.05, ***P < 0.001

## Data Availability

Data will be made available on request.
