# Supplementary figures and images for: A novel role of IGFBP5 in the migration, invasion and spheroids formation induced by IGF-I and insulin in MCF-7 breast cancer cells
Source: Breast Cancer Res Treat. 2024 Jun 19;208(1):79–88. doi: 10.1007/s10549-024-07397-5 (PMC11452427; doi:10.1007/s10549-024-07397-5)

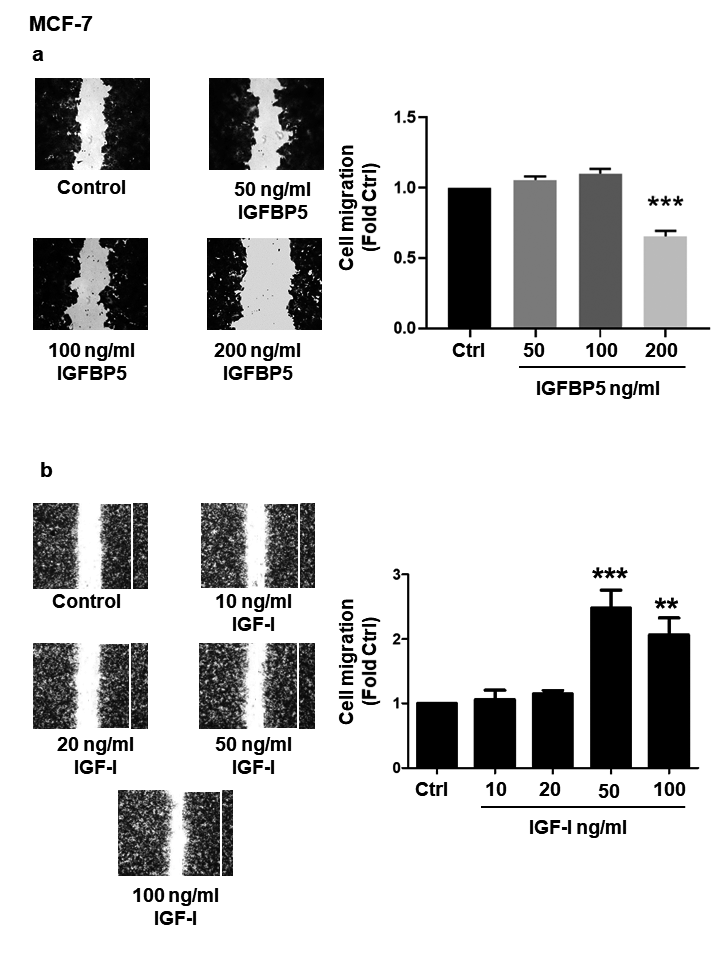

Supplement: Supplementary file 1 — Supplementary file1 (TIF 151 KB) Figure 1S Role of IGFBP5 and IGF-I in the migration of MCF-7 and MDA-MB-231 cells. a Confluent cultures of MCF-7 cells were scratched and treated with 50, 100 and 200 ng/ml IGFBP5 for 48 h. b Confluent cultures of MCF-7 cells were scratched and treated with 10, 20, 50 and 100 ng/ml IGF-I for 48 h. Graph is the mean ± S.D. and indicates the fold of migration above control value. **P < 0.01, ***P < 0.001 [file 10549_2024_7397_MOESM1_ESM.tif]

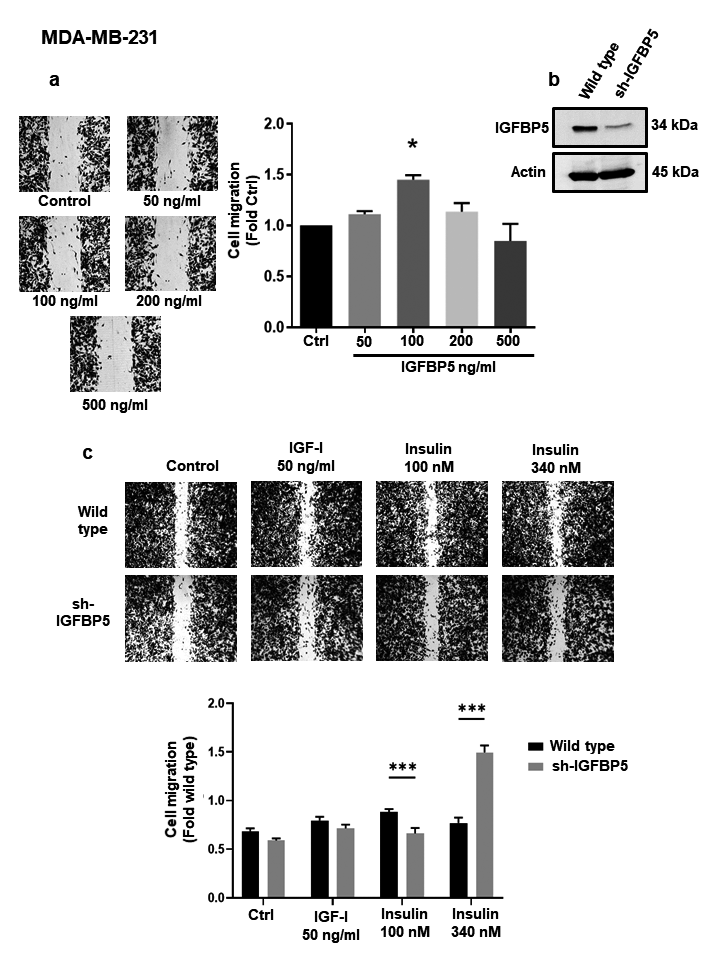

Supplement: Supplementary file 2 — Supplementary file2 (TIF 212 KB) Figure 2S IGFBP5 induces migration in MDA-MB-231 breast cancer cells. a Confluent cultures of MDA-MB-231 cells were scratched and treated with 50, 100, 200 and 500 ng/ml IGFBP5 for 48 h. b Lysates from wild type MDA-MB-231 cells and sh-IGFBP5 MDA-MB-231 cells were analyzed by WB with anti-IGFBP5 Ab and anti-actin Ab. c Confluent cultures of wild type MDA-MB-231 cells and sh-IGFBP5 MDA-MB-231 cells were scratched and untreated and treated with 50 ng/ml IGF-I, 100 and 340 nM insulin. Graphs are the mean ± S.D. and indicate the fold of migration above control value. *P<0.05, ***P < 0.001 [file 10549_2024_7397_MOESM2_ESM.tif]
